# Sera from breakthrough infections with SARS-CoV-2 BA.5 or BF.7 showed lower neutralization activity against XBB.1.5 and CH.1.1

**DOI:** 10.1080/22221751.2023.2225638

**Published:** 2023-07-12

**Authors:** Shuo Liu, Ziteng Liang, Jianhui Nie, Wei bo Gao, Xinyi Li, Li Zhang, Yuanling Yu, Youchun Wang, Weijin Huang

**Affiliations:** a Chinese Academy of Medical Sciences & Peking Union Medical College, Beijing, People’s Republic of China; b Changping Laboratory, Beijing, People’s Republic of China; c Division of HIV/AIDS and Sex-transmitted Virus Vaccines, Institute for Biological Product Control, National Institutes for Food and Drug Control (NIFDC), Beijing, People’s Republic of China; d Department of Emergency, Peking University People’s Hospital, Beijing, People’s Republic of China; e West China School of Basic Medical Sciences & Forensic Medicine, Sichuan University, Chengdu, People’s Republic of China

**Keywords:** SARS-CoV-2, neutralizing antibody, breakthrough infection, convalescence serum, BA.5/BF.7

## Abstract

From December 2022 to January 2023, SARS-CoV-2 infections caused by BA.5 and BF.7 subvariants of B.1.1.529 (Omicron) spread in China. It is urgently needed to evaluate the protective immune responses in the infected individuals against the current circulating variants to predict the future potential infection waves, such as the BQ.1.1, XBB.1.5, and CH1.1 variants. In this study, we constructed a panel of pseudotyped viruses for SARS-CoV-2 for the past and current circulating variants, including D614G, Delta, BA.1, BA.5, BF.7, BQ.1.1, XBB.1.5 and CH.1.1. We investigated the neutralization sensitivity of these pseudotyped viruses to sera from individuals who had BA.5 or BF.7 breakthrough infections in the infection wave of last December in China. The mean neutralization ID50 against infected variants BA.5 and BF.7 are 533 and 444, respectively. The highest neutralizing antibody level was observed when tested against the D614G strain, with the ID50 of 742, which is about 1.52-folds higher than that against the BA.5/BF.7 variant. The ID50 for BA.1, Delta, and BQ.1.1 pseudotyped viruses were about 2–3 folds lower when compared to BA.5/BF.7. The neutralization activities of these serum samples against XBB.1.5 and CH.1.1 decreased 7.39-folds and 15.25-folds when compared to that against BA.5/BF.7. The immune escape capacity of these two variants might predict new infection waves in future when the neutralizing antibody levels decrease furtherly.

## Introduction

As of 17 February 2023, the COVID-19 pandemic has caused more than 756 million infections and over 6.8 million deaths (https://covid19.who.int), which has posed an unprecedented impact on the global economy and people's lives. Vaccines and therapeutic monoclonal antibodies have been developed timely to counter this pandemic [1–3]. However, the virus mutation rate as a single-stranded RNA virus [4] and the widespread circulation of infection synergically contributed to the virus rapid evolution and enhancement of the probability of immune pressure. The virus evolution from Wuhan-Hu-1, B.1, Alpha, Beta, Gamma, Delta virus has led to the diverse immune escape patterns. Since the emergence of the Omicron variant, which has shown marked immune escape capabilities, an increasing number of breakthrough infection cases have been reported [5].

Before the Omicron variant appeared, the neutralization level of the vaccine immune serum against the previous VOCs (Alpha, Beta, Gamma, Delta) decreased by less than four times, with a 3.9-fold reduction compared to the reference 614G variant [6]. The mean neutralization ID50 of these sera against Omicron decreased significantly, which is about 8.4-folds when compared to the D614G strain [7,8]. When the neutralizing antibody of the population decreases to a certain level, the immune responses may become insufficient to protect the circulating strains, which might lead to a new infection wave. This round of infection last December in China is mainly caused by sub-variants of B.1.1.529 (Omicron) most of which were BA.5 and BF.7. The neutralizing antibody titres of these infected individuals against potential circulating strains XBB.1.5 and CH.1.1 which spread in other countries could cast a light on the possibility of the future infection wave [9].

## Results

There are 27 mutations on the RBD of Omicron variants (BA. 1, BA. 5, BF. 7, BQ.1.1, XBB.1.5, CH.1.1) (Figure S1). We tested the neutralizing sensitivities of D614G, Delta, BA.1, BA.5, BF.7, BQ.1.1, XBB.1.5, and CH.1.1 variants against the serum samples from individuals who had recently infected BA.5 or BF.7, who have been inoculated with three doses of inactivated vaccine. Sixty-three serum samples were employed in this study, which were collected at 4 weeks ± 1 week post breakthrough infections (Table S1). According to the COVID-19 Clinical and Surveillance Data report released by the China CDC, the predominant variant for Beijing and Tianjin are BF.7, for Jiangsu and Inner Mongolia are both BF.7 and BA.5, for the other provinces are BA.5. In this study, 18 serum samples were collected from Beijing and the remaining 45 serum samples from Guangzhou. So, we considered all the samples from Beijing to be from BF.7 infected individuals, and samples from Gangzhou are from BA.5 infections and have done comparisons for these two groups. When tested against the eight pseudotyped viruses, no significant differences were found between the two groups (Figure S2). In the following investigation, we took the 63 samples as a whole (BA.5/BF.7) to do the analysis.

**Figure 1.**
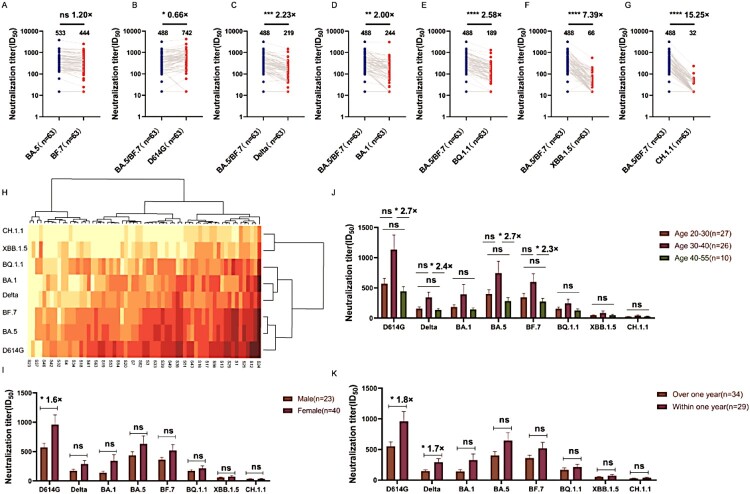
The neutralization capacities of convalescent sera from BA.5/BF.7 breakthrough infections against the pseudotyped viruse BF.7 (A) compared to BA.5, against the pseudotyped viruse D614G (B), Delta (C), BA.1 (D), BQ.1.1 (E), XBB.1.5 (F) and CH.1.1 (G) compared to BA.5/BF.7, The neutralization activity of 63 convalescent sera from COVID-19 patients was tested. The neutralization ID50 and decreased fold compared to the reference strain BA.5 or BA.5/BF.7 was also displayed as indicated. Data represented ID50 of three independent experiments. H. The heatmap of individual neutralization data. Data represented mean ID50 of three independent experiments. The red to yellow colour represented ID50 high to low as shown in the scale bar. S1–S63 sera were collected 1-month after recovery. The effects of gender (I), age (J), and the interval between the last vaccination and breakthrough infection (K) on the immune responses. *n* in the figure represents the number of samples.

BA.5/BF.7 breakthrough infection induced high neutralizing titres against D614G, BA.5, and BF.7. The GMTs (Geometric mean antibody titres) for D614G, BA.5, and BF.7 were all over 400. The ID50 of Delta, BA.1, and BQ.1.1 pseudotyped viruses decreased by about 2-3-folds. And the neutralization titres for XBB.1.5 and CH.1.1 decreased 7.39-15.25-folds compared to BA.5/BF.7 (Figure 1A–G). According to the heat map results, we can see that XBB.1.5 and CH.1.1 are both variants of BA.2.75, which have obvious effects on serum escape during the recovery period (Figure 1H). The subvariant XBB.1.5 has three additional mutation sites V445P, G446S, and F490S in the RBD domain, while the subvariant CH.1.1 only has one additional mutation site G446S in the RBD domain. And the XBB.1.5 and CH.1.1 share the mutation site G446S in the RBD domain, the neutralization titres for XBB.1.5 and CH.1.1 decreased 7.39-15.25-folds compared to BA.5/BF.7. The shared mutation site G446S in the RBD domain may play a key role for the dramatically decreased neutralization titres against the serum samples [9].

To study the effects of gender (Figure 1I), age (Figure 1J), and the interval between the last vaccination and breakthrough infection (Figure 1K) on the immune responses, we clustered the serum samples into different groups based on their background information (Table S1). When the serum samples were clustered into two groups by gender, relatively higher neutralizing antibody titres were observed in the samples from female individuals. Only when tested against the D614G pseudotyped virus, statistical significance was observed in the female group with 1.6-fold higher titres compared to the male group (Figure 1I). When it comes to age analysis, the individuals were divided into three groups: 20–30, 30–40, and 40–55. Significant differences between the 30–40 and 40–55 groups when tested against some specific pseudotyped viruses, including D614G, Delta, BA.5, and BF.7 (Figure 1J). By calculating the timing of the third dose of the vaccine, it has been determined that the shortest time between vaccination and infection is four months. Additionally, the majority of breakthrough infections occur six months or more after vaccination. When we analysed the effects of the interval between the last vaccination and breakthrough infection on the immune responses, we found the shorter the interval the higher the neutralization titres, among which significant differences were observed when tested against the pseudotyped viruses D614G and Delta (Figure 1K).

## Discussion

For the current epidemic of BA.5 and BF.7 variants in China, the serum samples from the recently infected persons have high neutralizing antibody levels. This result suggests that it may be able to prevent the secondary infection of BA.5 and BF.7 variants in the near future. The level of neutralizing antibody against the wild strain is the highest which may be attributed to the imprinted original immune responses against the prototype vaccine strain. This result suggests that the reinfection after vaccination can effectively boost the immune response. It should be noted that the neutralizing antibody level for XBB.1.5 and CH.1.1 variants is relatively lower with the ID50 only 32-66. The antibody titres in convalescent sera against the XBB 1.5 and CH 1.1 variants decreased to a significant extent, making it impossible for them to effectively neutralize these two variants. XBB.1.5 and CH1.1 share the same site mutation G446S, which might play a crucial role in the immune escape for the two variants. G446S has been reported to confer great resistance to a major class of neutralizing antibodies targeting at the right shoulder of RBD through altering microenvironments at the binding interface [12,13]. With the level gradually decreasing over time, XBB.1.5 and CH.1.1 variants may cause re-infection in these individuals infected in the last December infection wave. Before this breakthrough infection in China, the vaccination rate of infected persons in this round is 90.5% [14]. Although most people have been vaccinated with three doses of inactivated vaccines, the interval between vaccination and this breakthrough infection wave for most individuals were longer than one year. And the level of antibody in the body was insufficient to protect BA.5 and BF.7 variants, resulting in about 80% of COVID-19 infection rate in China [14]. The serum from individuals with breakthrough infection of the BF.7 strain can only protect the infected BA.5 variants (BF.7 and BQ.1.1), and cannot effectively neutralize the variants based on BA.2.75 (XBB.1.5 and CH.1.1) [3,13]. It suggests that although natural infection could improve the immune responses, the protective effect of variants that are antigenically distant from the infecting virus might not be effectively neutralized [15]. Therefore, it might be necessary to periodically boost the immune responses with antigens derived from the circulating variants to maintain the protective antibody level in the population. The limitation of this study was the small number of the serum samples. To investigate and compare the immune responses of the individuals from different gender groups, age groups, or intervals between vaccination and breakthrough infection groups, a large population cohort should be employed to mitigate the potential bias due to the sample selection.

In summary, this study has verified that the SARS-CoV-2 virus continues to evolve and evolve to evade the immune response to breakthrough infection, which sounds the alarm to the world and has important implications for public health planning and the development of matching strategies.

## Methods

### Pseudotyped SARS-CoV-2 variants

The Omicron variant gene of SARS-CoV-2 spike protein (GISAID: EPI_ISL_6590782.2) was optimized and synthesized using mammalian codons, and then cloned into pcDNA3.1 vector as described before [10]. Plasmids expressing S protein of D614G, Delta, BA.1, BA.5, BF.7, BQ.1.1, XBB.1.5, CH.1.1 SARS-CoV-2 variants were previously constructed [11]. The pseudotype SARS-CoV-2 variant based on VSV was transfected into 293 T cells (CRL-3216) with S protein expression plasmid and infected with G* Δ G-VSV (Kerafast, Boston). The titre of pseudovirus was evaluated by using Huh 7 (JCRB0403) cells through three times continuous dilution. Chemiluminescence signals were detected after cells and viruses were incubated at 37°C and 5% CO_2_ for 24 h. The Britellite Plus reporter gene assay system (PerkinElmer, Thermo Fisher Scientific) and the PerkinElmer Ensight photometer are utilized for signal acquisition. The detailed procedure has been described in our previous publication [10].

### *In vitro* neutralization assay

Briefly, add 100 μl of samples 96-well plate and the samples were 1:30 diluted, followed by a 3-fold serial dilution. The diluted samples were mixed with 50 μl pseudotyped SARS-CoV-2 variants (1.3 × 10^4^ TCID_50_) in 96-well plates, respectively. The mixture was followed by incubation at 37°C for 1 h, and then mixed with Huh 7 cells (2–3 × 10^4^ cells/well) followed by incubation at 37°C in a humidified atmosphere with 5% CO_2_. The chemiluminescence signals in terms of relative luminescence unit (RLU) value was determined as described previously. The 50% inhibition dilution (ID50) was calculated using the Reed–Muench method [10].

### Sera from SARS-CoV-2 convalescent patients

A total of 63 samples with the relative high neutralization activity in our preliminary study were selected. CS1–18 sera were collected 4 weeks ± 1 week after recovery from patients who had BA.5 or BF.7 breakthrough infection were collected by Institute for Biological Product Control, National Institutes for Food and Drug Control (NIFDC), Division of HIV/AIDS and Sex-transmitted Virus Vaccines, Institute for Biological Product Control.; CS19–63 sera were collected 4 weeks ± 1 week after recovery from Guangzhou Darui Biotechnology Co., Ltd. Consent forms were signed prior to blood collection.

According to the Helsinki Declaration, for the purpose of the study, we collected each participant's clinical information and blood samples and published the data generated by the study. We obtained their written informed consent. All authors have contributed to data collection, analysis, discussion, and interpretation of results. All authors have read and approved the final draft.

### Statistical analysis

Data were analysed with GraphPad Prism 8.0 software (GraphPad, San Diego, CA). The results are presented as the means § standard deviations (SD). Significance thresholds: *p < 0.05, **p < 0.01, ***p < 0.005, and ****p < 0.001.

## Supplementary Material

Supplemental Material
