# Supplementary material for: Sera from breakthrough infections with SARS-CoV-2 BA.5 or BF.7 showed lower neutralization activity against XBB.1.5 and CH.1.1
Source: Emerg Microbes Infect. 2023 Jul 12;12(2):2225638. doi: 10.1080/22221751.2023.2225638 (PMC10339773; doi:10.1080/22221751.2023.2225638)
Supplement: Supplemental Material [file TEMI_A_2225638_SM5134.docx]

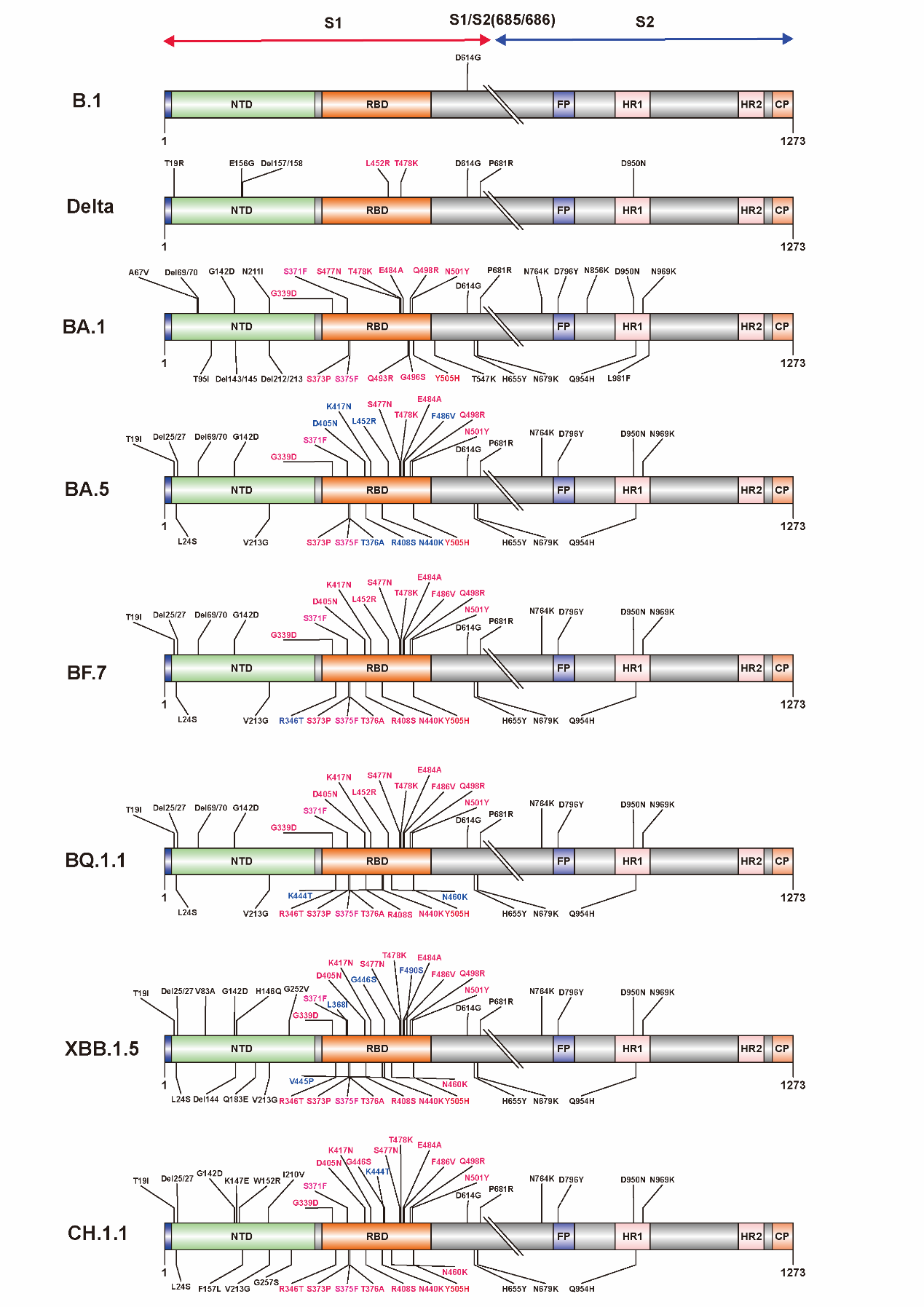


**Fig S1** Schematic diagram of the spike protein sequences for the Delta, BA.1,BA.5, BF.7, BQ.1.1, XBB.1.5 and CH.1.1 variants; mutations as compared with the B.1 spike protein are shown, The red colour shows the mutations of RBD, the blue colour shows more the mutations of RBD than the previous variant.

**Fig S2**. The comparison of the neutralizing capacity between the BF.7 and BA.5 serum samplses when tested against the pseudotyped viruses D614G, Delta, BA.1, BA.5, BF.7, BQ.1.1, XBB.1.5, and CH.1.1, n in the figure represents the number of samples.
